# Amyloid Fibrils Enhance the Topical Bio‐Adhesivity of Liquid Crystalline Mesophase‐Based Drug Formulations

**DOI:** 10.1002/adhm.202202720

**Published:** 2023-02-01

**Authors:** Francesca Damiani Victorelli, Camila Fernanda Rodero, Viviane Lutz‐Bueno, Marlus Chorilli, Raffaele Mezzenga

**Affiliations:** ^1^ Department of Health Sciences & Technology ETH Zurich Zurich 8092 Switzerland; ^2^ Department of Drugs and Medicine School of Pharmaceutical Sciences São Paulo State University Araraquara São Paulo 14800‐903 Brazil; ^3^ Paul Scherrer Institute (PSI) Villigen 5232 Switzerland; ^4^ Department of Materials ETH Zurich Zurich 8093 Switzerland

**Keywords:** amyloid fibrils, bio‐adhesiveness, inflammatory diseases, liquid crystalline mesophases, topical treatments

## Abstract

Despite their distinctive secondary structure based on cross *β*‐strands, amyloid fibrils (AF) are stable fibrous protein aggregates with features similar to collagen, one of the main components of the extracellular matrix, and thus constitute a potential scaffold for enhancing cell adhesion for topical applications. Here, the contribution of AF to skin bio‐adhesivity aiming toward topical treatments is investigated. Liquid crystalline mesophase (LCM) based on phytantriol is formulated, with the aqueous phase containing either water or a solution of 4 wt% amyloid fibrils. Then resveratrol is added as a model anti‐inflammatory molecule. The developed LCM presents a double gyroid Ia3d mesophase. The incorporation of AF into the LCM increases its bio‐adhesive properties. In vitro release and ex vivo permeation and retention confirm the controlled release property of the system, and that resveratrol is retained in epidermis and dermis, but is also permeated through the skin. All formulations are biocompatible with L929 cells. The in vivo assay confirms that systems with AF lead to a higher anti‐inflammatory effect of resveratrol. These results confirm the hypothesis that the incorporation of AF in the LCM increases the bio‐adhesiveness and efficiency of the system for topical treatment, and consequently, the therapeutical action of the encapsulated drug.

## Graphical abstract

Amyloid fibrils are stable aggregates that mimic the extracellular matrix and are a potent scaffold for cells. The incorporation of amyloid fibrils in topical drug delivery systems, such as liquid crystalline mesophases, increases their bio‐adhesive properties, solving the problem of fluidity of topical formulations and enhancing the treatment of topical diseases.

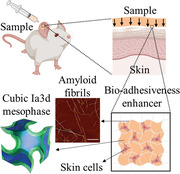

## 1 Introduction

Amyloid fibrils (AF) are stable aggregates of soluble globular proteins.^[^ ^1^, ^2^ ^]^ In vitro, their synthesis is promoted by concomitant heat denaturation and acid hydrolysis, which turn proteins into peptide sequences and promote self‐assembly of the latter. The spatial configuration assumed by the mentioned peptides is rich in *β*‐strands and *β*‐sheets, which are arranged perpendicular to and along the fibril axis, respectively.^[^ ^3^ ^]^ This specific arrangement makes AF the minimum energy state in the protein conformation landscape,^[^ ^4^ ^]^ and endows them with pronounced stability against degradation.^[^ ^1^, ^2^ ^]^ Moreover, in terms of unique microscopic features, AF also possess mesoscopic properties of interest, e.g., their few‐nm width, together with a contour length that spans in the µm range, make them objects with extremely high‐aspect and surface‐to‐volume ratios. These properties encouraged exploration of the use of AF in a broad range of applications. From water purification^[^ ^5^ ^]^ to the formation of liquid–crystalline phases,^[^ ^6^ ^]^ from the synthesis of food gels^[^ ^7^ ^]^ to the regulation of gut microbial dysbiosis,^[^ ^8^ ^]^ the breadth of fields that can benefit from research on AF is greatly expanding. Among these fields, biomedicine is expected to be particularly enriched by research on these proteinaceous aggregates: from past studies, AF are known to closely mimic the extracellular matrix (ECM) and to present some adhesivity.^[^ ^9^ ^]^

ECM is found in tissues and organs as a support for cells to adhere and grow, and it is mainly composed of collagen, fibronectin, and laminin. ECM forms a meshwork, with the main function of serving as a scaffold for cells.^[^ ^10^ ^]^ The adhesivity of cells to scaffolds is essential for the development of regenerative medical applications.^[^ ^11^, ^12^ ^]^ Some studies indicate that cells are able to adhere to AF. For example, Jacob and colleagues suggested that cells can attach to AF through lipid–fibril interactions, as well as integrin‐mediated ones. They showed that the cells that adhered to the AF surface used focal adhesion machinery to facilitate integrin‐mediated cell adhesions. This indicates that the interaction of cells and AF engage the integrin machinery for cell adhesion, similarly to ECM proteins.^[^ ^9^ ^]^ Reynolds and colleagues cultivated L929 fibroblast cells on the AF surface. They observed that the cells remained on the surface of the AF even after washing, and suggested that the nanotopography can influence integrin‐mediated cell adhesion, improving the cell–substrate interaction.^[^ ^13^ ^]^ If AF can be used as a scaffold for cell adhesion, these aggregates could also increase the bio‐adhesiveness and interaction between topical drug delivery systems and skin cells. Based on this hypothesis, here we investigated the ability of AF to increase the bio‐adhesive properties of topical drug delivery systems, such as liquid crystalline mesophases (LCM).

Liquid crystalline mesophases (LCM) are ordered self‐assembled structures capable of solubilizing lipophilic and hydrophilic drugs, protecting them from physical and chemical degradation, promoting controlled drug release,^[^ ^14^, ^15^ ^]^ and contributing to greater therapeutic efficiency. This system is widely explored for topical applications,^[^ ^16^, ^17^, ^18^, ^19^ ^]^ mainly for drugs that have low solubility, rapid metabolism, and low bioavailability, such as resveratrol (RES).^[^ ^20^, ^21^ ^]^

**Figure 1:**
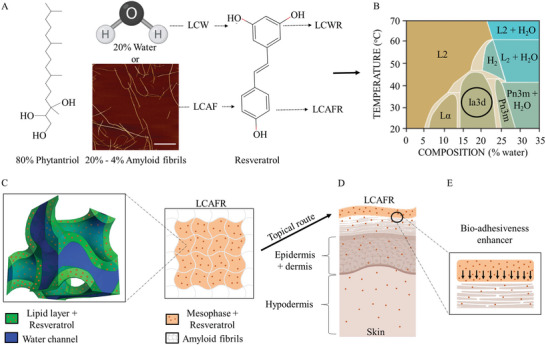
Study outline. A) Components of the liquid crystalline mesophase (LCM) composed of phytantriol with: water (LCW), water and resveratrol (LCWR), amyloid fibrils (LCAF), and amyloid fibrils and resveratrol (LCAFR). B) Phase diagram of phytantriol. Adapted with permission.^[^ ^40^ ^]^ Copyright 2020, American Institute of Physics. C) Cubic Ia3d mesophase loaded with resveratrol, which interacts with the AF. D) Scheme of topical application of LCAFR, and the delivery of resveratrol through the skin. E) Scheme of the enhanced bio‐adhesive property of LCAFR.

Resveratrol (RES) is a natural polyphenol found in some medicinal plants, which presents several pharmacological activities, such as anti‐cancer, anti‐bacterial, and anti‐inflammatory.^[^ ^22^, ^23^, ^24^, ^25^ ^]^ The anti‐inflammatory activity is related to its ability to inhibit cyclooxygenase enzymes, which are responsible for the production of pro‐inflammatory molecules.^[^ ^22^, ^26^ ^]^ Considering that RES is an effective inhibitor of cyclooxygenase activity, we explored the cutaneous anti‐inflammatory activity of RES incorporated into the liquid crystalline mesophase (LCM) with the addition of amyloid fibrils (AF) to improve bio‐adhesiveness (**Figure** **1**C–E).

Cutaneous treatment offers certain major advantages, such as minimizing side effects, avoiding the first‐pass effect, improving patient adherence to therapy, allowing self‐administration, and being more selective to a specific site. However, keeping the formulation attached to the desired surface during a time that is sufficiently long for the drug to be released is a challenge, since fluid formulations may easily run off, and the topical treatment can become inefficient. Therefore, strategies to improve the bio‐adhesiveness of the formulations, and to make them stable for contact with the skin for a long period of time, are essential to increase drug absorption and enhance the efficiency of the treatment.^[^ ^27^, ^28^, ^29^ ^]^

In the literature, studies used polymers, such as carbomers,^[^ ^30^, ^31^ ^]^ poloxamer^[^ ^32^ ^]^ and chitosan,^[^ ^33^ ^]^ to increase the bio‐adhesion of drug delivery systems. However, amyloid fibrils (AF) present several advantages compared to other bio‐adhesive materials:^[^ ^34^, ^35^, ^36^ ^]^ they are natural, present no potential harm to skin, and are inexpensive. AF can be produced from globular proteins of animal (milk)^[^ ^37^ ^]^ or vegetable origin (oat or soy).^[^ ^38^, ^39^ ^]^ Moreover, the production of AF is very simple: it involves only heating to mild temperatures of ≈90 °C, and lowering the pH to 2.^[^ ^2^ ^]^

As bio‐adhesive drug delivery systems can enhance the efficiency of topical treatment, we explored the bio‐adhesivity of AF from *β*‐lactoglobulin (*β*lg), as well as its potential to optimize topical drug delivery and to improve the efficiency of cutaneous treatment. We developed liquid crystalline mesophases (LCM) composed of phytantriol with water (LCW), water and resveratrol (LCWR), amyloid fibrils (LCAF), and amyloid fibrils and resveratrol (LCAFR) (Figure 1A). We characterized the structure by SAXS and the viscoelastic behaviour by rheology. We also evaluated the release and ex vivo permeation and retention profile of RES in porcine skin. Biocompatibility was analysed using L929 cell line. The enhancement of the therapeutic effect was verified using in vivo ear edema. The results showed that the incorporation of AF did not change the structure, rheological behaviour and release, permeation, and retention profile of RES. However, AF significantly increased the bio‐adhesiveness of the LCM. In the in vitro cytotoxicity assay, the LCM showed no toxicity to healthy cells. Finally, the RES‐loaded LCM with AF enhanced anti‐inflammatory activity in the skin. This study suggests that AF can impart bio‐adhesiveness to drug delivery systems and improve the effectiveness of topical treatment.

## 2 Results and Discussion

Amyloid fibrils (AF) are proteinaceous aggregates that present cell bio‐adhesion properties.^[^ ^9^, ^13^ ^]^ A solution of 4 wt% AF was used to prepare liquid crystalline mesophases (LCM) composed of phytantriol. Phytantriol is a biocompatible lipid that self‐assembles into LCM in the presence of water,^[^ ^40^, ^41^ ^]^ which is a potential drug delivery system.^[^ ^14^, ^15^, ^16^, ^42^ ^]^ It can form different mesophases,^[^ ^40^ ^]^ as shown in the phase diagram in Figure 1B. Here, we explore a new bio‐adhesive strategy: we trap the LCAFR in a network of AF (Figure 1C). There is a previous study from our group discussing the incorporation of the amyloids in hexagonal lipid mesophases: in that case the 1D symmetry of the columnar hexagonal phase is compatible with the highly rigid amyloid fibrils as shown by AFM imaging.^[^ ^43^ ^]^ In the present case, the isotropic nature of the bicontinuous cubic phase makes impossible to detect any orientation of the amyloids via any imaging technique. This, combined with the mismatch between the persistence length of amyloids, which is of the order of microns,^[^ ^44^ ^]^ with the curvature of the lipid bilayer, which is 3 orders of magnitude lower^[^ ^45^ ^]^ lead us to hypothesise that the amyloid fibrils in the present work locate essentially at the gran boundaries of the lipid mesophases considered. Moreover, regarding the incorporation of the drug, we suggest that resveratrol is incorporated into lipid bilayer composed of phytantriol, because this drug is lipophilic.^[^ ^46^ ^]^

As AF can serve as a scaffold for cells, it should also increase bio‐adhesion, and consequently the contact of the LCM with the biological surface, such as the skin (Figure 1E). With such approach, the contact between the drug delivery system and the skin could increase with the presence of AF, thus improving the treatment of topical diseases.

### 2.1 Structural Stability

**Figure 2:**
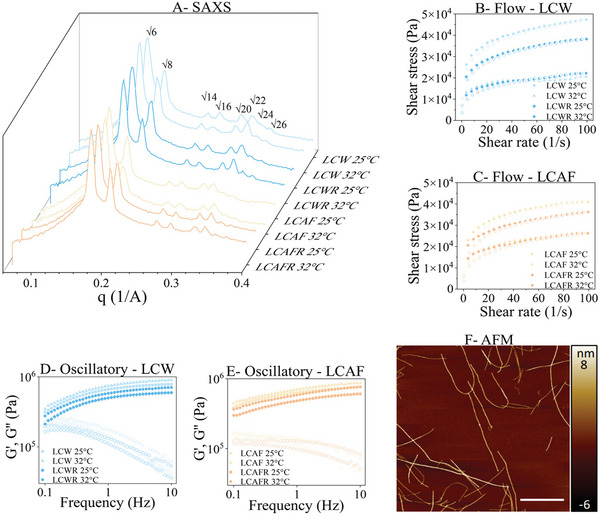
A) SAXS curves of LCW, LCWR, LCAF, and LCAFR at 25 and 32 °C. B) Flow rheograms of LCW and LCWR at 25 and 32 °C. (C) Flow rheograms of LCAF and LCAFR at 25 and 32 °C. The closed symbols mean the up‐flow curves, and the open symbols mean the down‐flow curves. D) Frequency sweep profile of the storage modulus *G*′ (closed symbols) and the loss modulus *G*′′ (opened symbols) of the LCW and LCWR at 25 and 32 °C. E) Frequency sweep profile of the storage modulus *G*′ (closed symbols) and the loss modulus *G*′′ (opened symbols) of the LCAF and LCAFR at 25 and 32 °C. F) AFM image of *β*lg fibrils in water (pH 2). The scale bar is of 1 µm.

**Figure** **2**A describes the structure of unloaded liquid crystalline mesophases with water (LCW) or amyloid fibrils (LCAF) and resveratrol‐loaded liquid crystalline mesophases with water (LCWR) or amyloid fibrils (LCAFR) at room temperature (25 °C) and human skin temperature (32 °C). The scattering of the samples presented gyroid cubic mesophase (Ia3d) at both temperatures. The Ia3d unit lattice parameters estimated for LCW, LCWR, LCAF, and LCAFR at 25 °C were 93.47, 91.20, 94.00, and 88.00 Å, respectively, and at 32 °C were 92.32, 90.11, 89.59, and 85.99 Å, respectively. We observed that when the temperature increased, the lattice parameter of the mesophases decreased. This change is caused by the increase in the curvature of lipid bilayer with increasing temperature.^[^ ^47^ ^]^ Figure 2F shows the atomic force microscopy (AFM) result that confirms the formation of amyloid fibrils that were incorporated into the liquid crystalline mesophases (LCAF and LCAFR).

We evaluated the rheological behavior of the liquid crystalline at the same temperatures. The thixotropic behavior of the mesophase samples is shown in Figure 2B,C, and it is found that they are pseudoplastic (*n* < 1). Pseudoplastic flow indicates that the viscosity of the sample decreases when the shear rates increase, since shear‐thinning occurs due to the reorientation of the structures in the direction of the flow.^[^ ^46^ ^]^ Moreover, the descending curve returned below the ascending curve, indicating the thixotropic behaviour. This behaviour suggests that the viscosity of the samples are shear‐dependent, i.e., when the shear stress is removed, the original structure of the LCM is recovered.^[^ ^27^, ^46^ ^]^ Pseudoplasticity and thixotropy are important characteristics for topical drug delivery systems, since the decrease in viscosity during application facilitates their spreading. After application, the formulations could then return to their original viscosity, allowing better adherence to the skin. The oscillatory analysis in Figure 2D,E indicates that the LCM are stable, since G′ > G″, and therefore behave like a gel with organized structures.^[^ ^46^, ^48^, ^49^ ^]^ The results also show that the incorporation of AF, resveratrol (RES), and temperature change did not affect the internal nanostructures or the rheological behavior of the LCM.

### 2.2 Bio‐Adhesive Properties of Amyloid Fibrils, and Release and Ex Vivo Permeation and Retention Studies

Amyloid fibrils (AF) mimic the extracellular matrix, an integral component of organs that provides physical stability to tissues,^[^ ^10^ ^]^ and can be the basis for cell attachment via integrins.^[^ ^9^, ^13^ ^]^ Therefore, we investigated if AF could increase the bio‐adhesion of the liquid crystalline mesophase and alter the profile of RES release, permeation, and retention in the skin.

**Figure 3:**
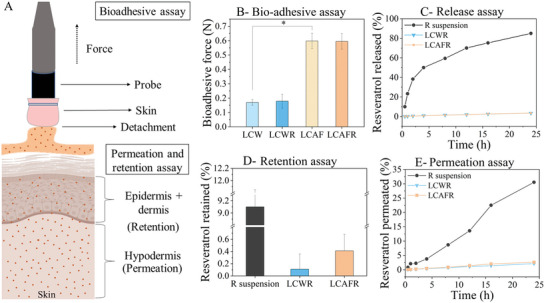
A) Schematic of the bio‐adhesive and permeation and retention assay. B) Bio‐adhesive force of the LCW, LCWR, LCAF, and LCAFR at 32 °C. The results are expressed as an average of five measurements, and the error is reported as standard deviation. C) In vitro release profile (%) of RES from LCWR, LCAFR, and RES suspension (*R* suspension) through a 0.45‐µm polyethersulfone membrane. D) Ex vivo retention (%) in epidermis + dermis through porcine skin of RES from LCWR, LCAFR, and RES suspension (*R* suspension). E) Ex vivo permeation (%) of RES from LCWR, LCAFR, and RES suspension (*R* suspension) through porcine skin.

**Figure** **3**B shows that we measured higher bio‐adhesive forces for the LCM containing AF compared to the LCM containing only water. Furthermore, the incorporation of RES did not change the bio‐adhesive profile of the systems. This result indicates that AF increased the interaction of the LCM with the skin, since the texture analyzer had to exert more force to detach the sample from the surface (Figure 3A).

Release studies were performed to evaluate the release profile of RES from LCWR and LCAFR, and from an aqueous suspension through a 0.45 µm polyethersulfone membrane. In Figure 3C, it can be seen that the release of RES was more controlled for LCWR and LCAFR when compared to the RES suspension, which already released 50% of the drug in the first 4 h. The release of RES from LCWR and LCAFR is very similar, 3.44% and 3.49%, respectively, and thus the network of AF does not hinder the release of the drug.

The retention and permeation of the RES incorporated in the liquid crystalline mesophase were investigated using porcine skin as a model (Figure 3A), and RES in suspension was employed as the control. The retention study showed that the amount of RES from the RES suspension and RES‐loaded LCM that could be retained in the stratum corneum was insignificant. However, the RES amount retained in the epidermis and dermis was ≈9.10% for RES suspension, and 0.11% and 0.41% for LCWR and LCAFR, respectively (Figure 3D). In addition, the RES in suspension and incorporated in the LCM permeated the skin (Figure 3E). RES in suspension permeated more since a higher amount of the drug is free. Contrarily, the RES incorporated in the drug delivery system must first be released from the system and then permeate the skin, thus corroborating the results of the release study. This result indicates that RES in suspension and incorporated in the LCM could permeate the skin, and that the drug will reach the blood circulation and have a systemic effect. While it increased the adhesiveness, the incorporation of AF did not significantly change the release, retention, and permeation profiles of the drug systems for topical applications.

AF present bio‐adhesion properties when in contact with the skin, and may be used in several topical drug delivery systems to increase not only the contact time of the sample to the skin, but also the drug absorption.^[^ ^16^ ^]^ Furthermore, the LCM with AF is promising for topical administration, since the RES released from LCAFR was not only retained, but also permeated through the skin. This profile suggests that the treatment may present a topical and systemic effect, thus improving the therapeutic effect.

### 2.3 Evaluation of Cytotoxicity by the Agar Diffusion Method and In Vivo Anti‐Inflammatory Activity

**Figure 4:**
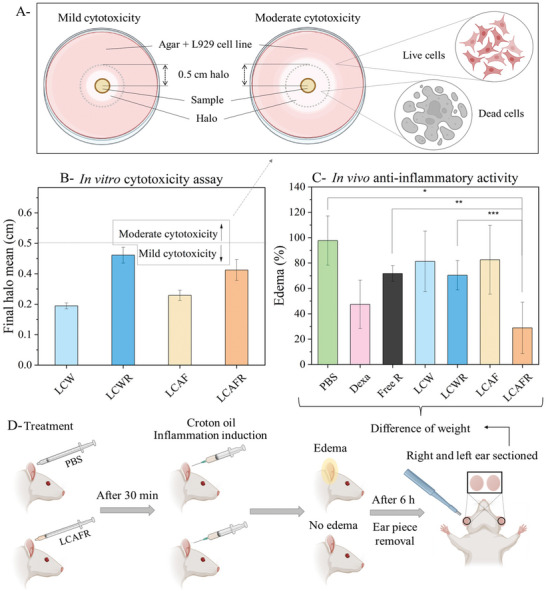
Cytotoxicity assessment by L929 cell line and anti‐inflammatory activity of the formulations. A) Scheme of the in vitro agar diffusion methodology. (B) Cytotoxicity halos obtained for LCW, LCWR, LCAF, and LCAFR in L929 cell line. Data are shown as mean ± SD of three individual experiments. C) In vivo anti‐inflammatory activity of PBS (negative control), dexamethasone (positive control), free RES, LCW, LCWR, LCAF, and LCAFR. Data are shown as mean ± SD of six individual experiments. D) Scheme of the in vivo ear edema assay methodology.

To assure that AF is not toxic and therefore safe to apply to the skin, we performed a cytotoxicity analysis. The cytotoxicity of the LCW, LCAF, LCWR, and LCAFR formulations was verified in vitro using the cell line L929, which is a healthy skin cell, by the agar diffusion method (**Figure** **4**A). The use of healthy skin cells is crucial to ensure that the formulation will present a safe pharmacological response. Figure 4B shows the mean sizes of the cytotoxicity halos, which represent dead cells (Figure 4A), obtained for each formulation. The table of degrees of cytotoxicity was used to obtain qualitative results. The negative control, which is a treatment that is not expected to produce results, was composed only of culture medium and did not show halo formations; therefore, it was classified as having 0 degree of cytotoxicity (absence). The positive controls (Triton‐X), which is a treatment that is known to produce results, exhibited halo formations greater than 1.0 cm and caused severe cytotoxicity. Our formulations showed mild cytotoxicity for L929 cells, since the halo sizes were smaller than 0.5 cm. Therefore, we suggest that the LCM with and without AF are not toxic to healthy cells. We confirm that the formulations do not damage skin cells, and thus they may be safely applied to the skin. Furthermore, we confirm that AF are non‐toxic for the skin cells, and that they could act as a safe bio‐adhesive enhancer.

After proving that it is safe, the in vivo assay (Figure 4C,D) verified the influence of amyloid fibrils (AF) in the anti‐inflammatory effect of resveratrol (RES) when incorporated in the liquid crystalline mesophases (LCM). The groups tested in the mice were LCW, LCAF, LCWR, LCAFR, and free RES. Dexamethasone was used as a positive control, and the animals that received phosphate‐buffered saline (PBS) were considered as the negative control. In Figure 4C, it can be seen that the group that was treated with RES incorporated in LCAF showed a greater decrease in edema than the groups treated with LCWR, free RES, and the negative control. This result is due to the bio‐adhesive property of AF that was confirmed in the bio‐adhesion assay. Since AF improves the bio‐adhesiveness of the LCM in the skin, a greater amount of RES was able to be in contact with the skin of the mice for a longer period of time. Therefore, the anti‐inflammatory treatment was significantly more efficient, decreasing the inflammation induced in the mice's ears. This result confirms that the incorporation of AF in drug delivery systems may enhance the treatment of topical diseases.

## 3 Conclusion

Amyloid fibrils (AF) are composed of proteins that promote cellular bio‐adhesion.^[^ ^9^, ^13^ ^]^ However, to the best of the authors’ knowledge, their bio‐adhesiveness to the skin has never been investigated. Here, we confirm that the incorporation of AF into liquid crystalline mesophase (LCM) improves its bio‐adhesion to skin. Conversely, AF did not influence the structure of the LCM, thus preserving the Ia3d mesophase. The rheological property of the LCM was also preserved, since the flow analysis showed pseudoplastic and thixotropic behavior, and the oscillatory analysis showed gel characteristics. The release, retention, and permeation profile of resveratrol (RES) incorporated into the LCM allowed the controlled release, and both retention and permeation, of RES in the skin. The LCM presented biocompatibility with healthy cells, as shown in the in vitro assay. The in vivo assay indicated that the liquid crystalline mesophases with amyloid fibrils (LCAFR) caused greater anti‐inflammatory activity than the LCM without AF. Based on these results, we can conclude that AF enable better interaction between the LCM and the skin, thus improving the effectiveness of the treatment, since more RES remains in contact with the skin for a longer period of time. Finally, this study supports our hypothesis that AF are a promising natural bio‐adhesion enhancer for drug delivery systems for topical administration, and that they may increase the efficiency of treating topical diseases.

## 4 Experimental Section

### Materials

The whey protein isolate (WPI) was supplied by Fonterra (New Zealand). Phytantriol (PHY) > 99.5% was purchased from DSM (Switzerland). Acetonitrile (HPLC grade) was purchased from Dinâmica (Brazil), and methanol 99.8% was purchased from Sigma Aldrich (Brazil). Sodium lauryl sulfate (SLS) 99.5% was purchased from Impex (Brazil). Resveratrol 99% was purchased from Galena (Brazil). All solutions were prepared using Milli‐Q water (18.2 MΩ cm^−1^; Millipore, USA).

### Preparation of Amyloid Fibrils

Amyloid fibrils (AF) were prepared via heat treatment at acidic conditions of whey protein isolate: during the process, the main protein fraction of WPI, namely *β*‐lactoglobulin, denatures and subsequent self‐assembly to fibrils, according to a previously developed protocol^[^ ^37^ ^]^ with slight adaptations. Firstly, 4 wt% of whey protein isolate was dispersed in Milli‐Q water. By adding citric acid, the solution's pH was adjusted to 2, followed by incubation of the solution at 90 °C for 5 h under constant stirring of 300 rpm. Afterward, the solution was quenched in an ice bath to stop the fibrilization process, and the formation of AF was confirmed through cross‐polarized light.

### Preparation of Liquid Crystalline Mesophase

Liquid crystalline mesophases (LCM) were obtained by mixing an oily phase (80%), composed of a phytantriol and aqueous phase (20%), composed of water (LCW) or dispersion of amyloid fibrils (LCAF), according to **Table** **1**. Resveratrol (RES) (2.5% w/w) was added to the oily phase and sonicated for 10 min to solubilize, and then the aqueous phase was immediately added.

**Table 1:** Nomenclature and composition of formulation

Formulation	Phytantriol	Water	Amyloid fibrils 4%	Resveratrol
LCW	80%	20%	–	–
LCAF	80%	–	20%	–
LCWR	77.5%	20%	–	2.5%
LCAFR	77.5%	–	20%	2.5%

### Small‐Angle X‐Ray Scattering

Small‐angle X‐ray scattering (SAXS) measurements were performed to determine the mesophase of the liquid crystalline system, using a Bruker AXS Micro instrument with a microfocused X‐ray source, of wavelength *λ*Cu K*α* = 1.5418 Å, operating at 50 kV and 1000 µA. Diffracted X‐rays signal was collimated by a 2D Kratky collimator and collected by a 2D Pilatus 100 K detector. The scattering vector *q* = (4 *π*/*λ*) sin *θ*, with 2*θ* being the scattering angle, was calibrated using silver behenate. Data were collected and azimuthally averaged using the Saxsgui software to produce 1D intensity versus scattering vector *q*, with a *q* interval of 0.004 to 0.4 Å^−1^. The measurements were performed at 25 °C, which was the room temperature considered for the administration of the formulation to the patient and at 32 °C, which is the temperature of the skin. The scattering intensity was collected for 20 min.

### Rheological Analyses

Rheological analyses were performed in triplicate at 25 and 32 °C using an AR2000 rheometer (TA Instruments, USA). For the flow analysis, a 20‐mm cone plate geometry and sample gap of 250 µm was used. The shear rate increased from 0.01 to 100 s^−1^, and then decreased back to 0.01 s^−1^. The flow index was defined according to the equation *τ* = *k* · *γ* ^*η*^, where *τ* represents the shear stress (Pa), *k* the consistency index (Pa s^n^), *γ* the shear rate (1/s), and *η* the flow index. For the oscillatory analysis, a 40‐mm parallel plate geometry and a sample gap of 500 µm was used. The linear viscoelastic region by stress sweep with a shear stress from 0.01 to 15 000 Pa and a frequency of 1 Hz was used. Afterward, the frequency sweep test was performed with a constant shear stress of 80 Pa to define the storage modulus (*G*′) and loss modulus (*G*″).^[^ ^46^ ^]^

### Ex Vivo Bio‐Adhesion Studies

The bio‐adhesive force between the porcine skin and the samples using the TA‐XT Plus texture analyzer (Stable Micro Systems, England) was evaluated. The porcine skin was prepared as described by Victorelli et al. (2018)^[^ ^33^ ^]^ and attached to the probe with a rubber ring. The formulations were placed under the probe at 32.0 ± 0.5 °C. Then, the probe was lowered at a speed of 1 mm/s until contacting the formulation. The formulation and the skin were kept in contact for 60 s, and then the probe was drawn upward at a speed of 0.5 mm s^−1^ until the formulation was detached from the skin. All formulations were tested five times.

### Release Studies and Ex Vivo Permeation and Retention Studies

A Microette (USA) consisting of Franz diffusion cells was used to perform the release and ex vivo permeation and retention studies of the control composed of a suspension of resveratrol (RES) in PBS and sodium lauryl sulfate (SLS), and RES incorporated into the liquid crystalline mesophases (LCM). In these experiments, a receptor solution composed of PBS pH 7.4 with 1% of sodium lauryl sulfate at 32 °C was used with adaptations according to Prezotti et al. (2018)^[^ ^50^ ^]^ and under stirring at 300 rpm. The samples were placed on the 0.45 µm polyethersulfone membrane or porcine skin, which was prepared as described by Victorelli et al. (2018),^[^ ^33^ ^]^ at the donor compartment of the Franz diffusion cell. The released and permeated RES were collected automatically after 30 min, 1, 2, 4, 8, 12, 18, and 24 h. The amount of RES released or permeated from the samples was quantified using high performance liquid chromatography (HPLC, Agilent, Japan) with a wavelength of 307 nm, performed under the following conditions: column type C18, 4.6 mm × 250 mm (Symmetry, USA) 5 mm at 32 °C. The mobile phase consisted of water and acetonitrile (65:35, v/v) and a flow rate of 1 mL min^−1^. The run time was set at 10 min, and the injection volume used was 10 µL. The extraction of the RES retained in the porcine skin was carried out using the method from Victorelli et al. (2018).^[^ ^33^ ^]^ After 24 h of the ex vivo permeation assay, the skin was cleaned using soft paper, and cut with scissors. The obtained fragments were placed in centrifuge tubes (Falcon, BD, USA) containing 4.0 mL of methanol and homogenized in Turrax at 10000 rpm for 2 min. Then, the tubes were centrifuged, and the RES present in the supernatant was quantified by HPLC using the same method described above and through the calibration curve in methanol.

### Evaluation of Cytotoxicity by the Agar Diffusion Method

L929 cell line in DMEM with 10% fetal bovine serum (FBS) at 37 °C with 5% CO_2_ was cultivated. A cell suspension with a concentration of 2.5 × 10^5^ cell mL^−1^ was pipetted into each well in six‐well plates and cultivated for 48 h. Afterward, the culture medium was removed, the wells were washed with PBS pH 7.4, and 1.8% agar medium with 0.01% neutral red dye and DMEM (1:1, v/v) was added. Sterile paper disks were embedded with the formulations and placed at the center of the well containing solidified agar.^[^ ^51^ ^]^ The halo formed with a pachymeter was measured.^[^ ^46^ ^]^ All formulations were tested in triplicate.

### Evaluation of the In Vivo Anti‐Inflammatory Activity of the Resveratrol Incorporated into the Liquid Crystalline Mesophases

The experimental protocol was performed in accordance with the animal experimentation ethics committee of the School of Pharmaceutical Sciences, São Paulo State University (protocol CEUA number 30/2021) and governmental and international guidelines on animal experimentation. The anti‐inflammatory activity of the samples was evaluated according to the croton oil‐induced edema ear model.^[^ ^52^ ^]^ Mice were divided into seven groups (six per group): group I mice received PBS (negative control); group II mice received topical dexamethasone (positive control); group III mice received free resveratrol (RES) dissolved in grape seed oil; group IV mice received LCW formulation; group V mice received LCWR formulation; group VI mice received LCAF formulation; and group VII mice received LCAFR formulation. The treatments were applied topically in the right ear of the mice 30 min before the induction of inflammation with the application of 20 uL of croton oil (2.5% [v/v] diluted in acetone) (Figure 4D). After 6 h of croton oil administration, the animals were euthanized. The left ear remained untreated and without inducing inflammation. Subsequently, the right and left ears were sectioned in circles of 6.0 mm in diameter. The effect of the treatments was evaluated according to the weight of the ears. The edematous response was expressed as the difference in weight between the circumference of the right and left ears. Anti‐inflammatory activity was expressed as the percentage of edema reduction of the treated ear compared to the untreated ear without induced inflammation.

### Statistical Analysis

The significance of the AF on bio‐adhesion, retention, and in vivo experiments was analyzed using two‐way ANOVA. Multiple comparisons between groups were determined using the Tukey test. These analyses were performed using OriginPro 2021 software. The analyses with *p*‐values <0.05 at a 95% confidence interval were considered significantly different.

## Conflict of Interest

The authors declare no conflict of interest.

## Data Availability

The data that support the findings of this study are available from the corresponding author upon reasonable request.
